# Assembly-History Dynamics of a Pitcher-Plant Protozoan Community in Experimental Microcosms

**DOI:** 10.1371/journal.pone.0042651

**Published:** 2012-08-07

**Authors:** Kohmei Kadowaki, Brian D. Inouye, Thomas E. Miller

**Affiliations:** Department of Biological Science, Florida State University, Tallahassee, Florida, United States of America; Argonne National Laboratory, United States of America

## Abstract

**Background:**

History drives community assembly through differences both in density (density effects) and in the sequence in which species arrive (sequence effects). Density effects arise from predictable population dynamics, which are free of history, but sequence effects are due to a density-free mechanism, arising solely from the order and timing of immigration events. Few studies have determined how components of immigration history (timing, number of individuals, frequency) alter local dynamics to determine community assembly, beyond addressing when immigration history produces historically contingent assembly.

**Methods/Findings:**

We varied density and sequence effects independently in a two-way factorial design to follow community assembly in a three-species aquatic protozoan community. A superior competitor, *Colpoda steinii,* mediated alternative community states; early arrival or high introduction density allowed this species to outcompete or suppress the other competitors (*Poterioochromonas malhamensis* and Eimeriidae gen. sp.). Multivariate analysis showed that density effects caused greater variation in community states, whereas sequence effects altered the mean community composition.

**Conclusions:**

A significant interaction between density and sequence effects suggests that we should refine our understanding of priority effects. These results highlight a practical need to understand not only the “ingredients” (species) in ecological communities but their “recipes” as well.

## Introduction

Community assembly has been a prominent concept in ecology; a variety of sometimes divergent views have reflected different assumptions and a confusing array of terminology. At one extreme, communities have been viewed as the product of random dispersal events, after which deterministic species sorting overrides immigration history. For example, Diamond [1] outlined a set of “assembly rules” of limited membership for the local fauna of bird communities in New Guinea that set limits on which species from the regional source pool could coexist. At the other extreme, the final community structure can be viewed as a historical artifact of the precise order of species' arrival. Although not supporting such an extreme role for historical contingency, Drake [2], used aquatic microcosms to show that community assembly depends in potentially complex ways on the identities and sequence of arrival of species as communities develop.

Empirical efforts to understand historical forces driving community assembly have included observational comparisons of natural communities at different localities at various disturbance levels (see, e.g., Urban [3], Weslien et al. [4]) and experimental perturbations of naturally recovering communities [5]–[7]; these empirical studies complement theoretical investigations into alternative stable states (e.g., Shurin et al. [8]) and transient states [9]. Communities from a wide range of habitats have been shown to be affected by the direct manipulation of immigration history (e.g., acacia ants, by Palmer et al. [10]; amphibians by Wilbur and Alford [11]; aquatic protists by Robinson and Dickerson [12] and Fukami [13]; ectomycorrhizal fungi by Kennedy et al. [14]; drosophilids by Shorrocks and Bingley [15]; wood-decaying fungi by Fukami et al. [16]). Along with empirical insights, theoretical work suggests that the context in which communities assemble can be altered by regional factors (e.g., large regional species pools, low rates of connectivity) and local factors (e.g., high productivity and low disturbance) [17]. These studies have explored aspects of the effects of immigration history on historically contingent assembly, but do not separate how various components of immigration history (timing, number of individuals, frequency) alter local dynamics to determine community assembly. No empirical studies have rigorously identified mechanisms by which local dynamics interact with immigration history.

History drives community assembly by two potentially independent mechanisms, density effects and sequence effects. Density effects are predictable dynamics that follow directly from different initial abundances of competitors and the time for unimpeded growth between colonizing events. For example, simple Lotka-Volterra models predict that, when conditions for a stable two-species equilibrium occur, communities will reach the same final equilibrium state regardless of the initial abundances of species, but when parameters create an unstable equilibrium, differences in species' abundances at the time when later colonists arrive determine which species outcompetes the other [18]. Density effects are independent of the history of other species and are firmly anchored in population-dynamics principles.

In contrast, sequence effects occur through differences that are unrelated to density but are due purely to the order in which species arrive. Possible mechanisms of sequence effects would include delayed life-history effects [19] and ecosystem engineering that alters fitness landscapes of competing species [20]. Note that, by our definitions, the widely used term “priority effects” (sensu Wilbur and Alford [11], Young et al. [21]) confounds density and sequence effects, even though theory gives reason to suspect that density and sequence effects on community assembly can differ (cf. Lotka [18], Connell and Slatyer [22]). Our separation of density and sequence effects is therefore essentially a claim that we should refine interpretations of priority effects. Previous experimental studies (e.g., Drake [2], Fukami [9], Robinson and Dickerson [12], Kennedy et al. [14], Collinge and Ray [23],) have shuffled the sequence of species introduction, but because they did not factorially vary the intensity of immigration (density of species) crossed with sequence of arrival, the underlying mechanisms leading to historically contingent community structure remain undetermined.

We varied density and sequence effects independently in a two-way factorial design to follow community assembly of an inquiline protozoan community in experimental microcosms. The community originates from the water-filled leaves of the purple pitcher plant, *Sarracenia purpurea*; in this ecosystem, energy is derived from allochthonous material in the form of insects that fall into the water-filled leaves and drown [24]. Bacteria make up the bottom trophic level as communities develop through immigration of protozoans, rotifers, and top predators [25]. This well-studied community has rapid dynamics and is ideal for studying assembly. We specifically tested the hypothesis that density and sequence effects interact to determine the mean and the variation (i.e., beta diversity) in community structure of protozoans in experimental microcosms.

## Materials and Methods

### Ethics statement

No specific permits were required for the described field and laboratory studies, as protozoa and bacteria sampling is freely allowed in natural areas in the Apalachicola National Forest. None of our studies involved endangered or protected species.

### Study organisms

*Poterioochromonas malhamensis* (species A), *Colpoda steinii* (species B), and Eimeriidae gen. sp. (species C) are protozoans commonly found in water-filled leaves of the purple pitcher plant, *Sarracenia purpurea,* in the Apalachicola National Forest, Florida, USA. The first is a suspension feeder; the latter two use both suspension feeding and grazing [26]. All three are generalist bacterivores and compete with each other for shared food in pitcher leaves and in experimental microcosms. We make the simplifying assumption that bacterial population dynamics are fast enough relative to protozoan dynamics that one can, without too much error, model the consumer-resource dynamics in a reduced phase space that considers only consumer protozoans (“ecological abstraction” sensu Schaffer [27]).

### Experiment design and procedure

**Figure 1:**
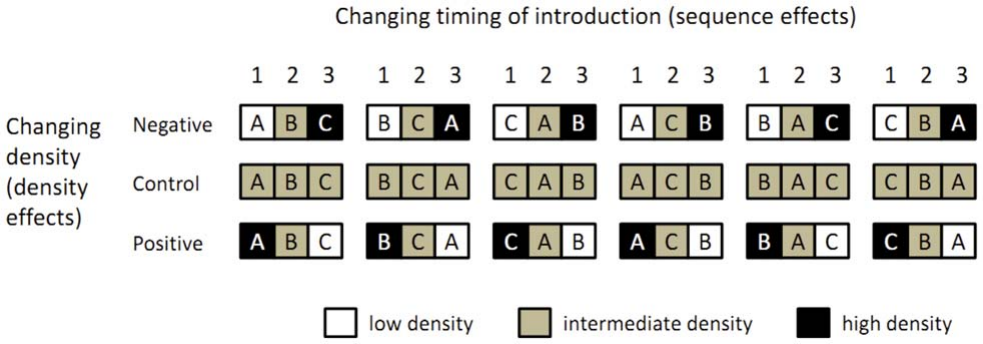
Experimental design for microcosms of the three protozoan species. Cells represent first, second, and third introductions (from left to right) of species into microcosms at 12-hour intervals; shading indicates low, intermediate, and high densities of protozoa in the aliquots introduced. Species A, *Poterioochromonas malhamensis*; species B, *Colpoda steinii;* species C, Eimeriidae gen. sp.

**Figure 2:**
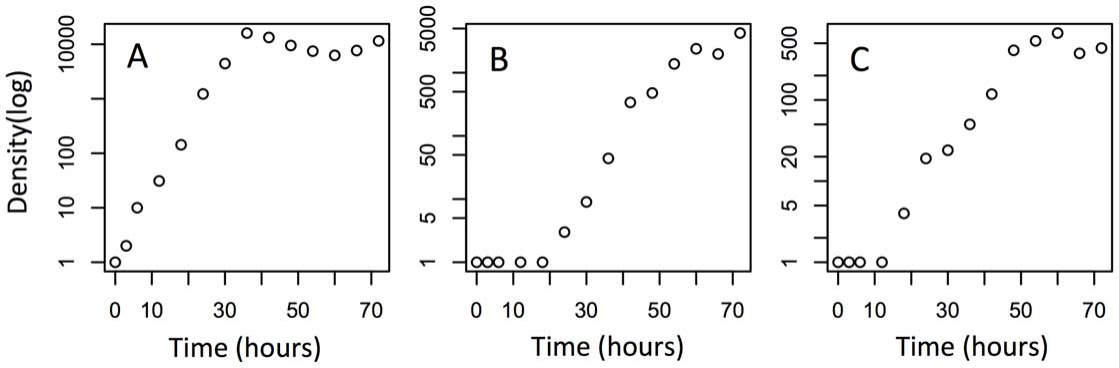
Single-species population growth curves of the three protozoan species used in the community-assembly experiment. A, B, and C as in Figure 1.

We assembled three-species protozoan communities in 10-mL microcosms, created as described below, by adding 0.50-mL aliquots from appropriate stock monocultures sequentially in a two-way factorial design (3 density levels×6 sequence levels), replicated in three temporal blocks, each of which contained 18 microcosms. Successive blocks were started at 1-hour intervals (i.e., 54 microcosms were started over 3 hours). Sequence treatment included six categorical levels, corresponding to all possible orders for introduction of the three species (Fig. 1). For each density treatment, we produced three scenarios, in which introduction density was negatively correlated, positively correlated, and uncorrelated with the order of introduction (Fig. 1). A negative correlation would result in stronger density effects of early-arriving species on later-arriving species, whereas a positive correlation would be expected to produce weaker density effects of early species on later species. Lack of correlation was intended as a control. A preliminary study showed that single-species populations of the three species readily attain quasi-steady states within 72 hours under the same immigration density setting (Fig. 2) and allowed us to estimate microcosm equilibrium carrying capacities for each species. Differences in maximum population density (carrying capacity) among the three species possibly reflect differences in body size. The immigration densities and times of introduction were therefore determined so that initial high-, intermediate-, and low-density introductions of the first colonizer result in its having 100, 50, and 25% of equilibrium density at the time of the introduction of the third species.

Microcosms initially contained 10 mL (within the range of natural leaf pitcher volumes) of sterile water that was inoculated with bacteria by addition of 1.6 mg of Tetramin fish food (Tetra Werke, Germany) and exposure to open air for 24 hours before the introduction of protozoan species. The first, second, and third species were added from stock monocultures after 0, 12, and 24 hours, respectively. Differences in introduction density were created by dilution of high-density stock cultures with medium similarly inoculated with bacteria; Table 1 gives the densities of the three protozoan species in the 0.50-mL aliquots used for introduction. After the initial 24 hours, 0.10-mL subsamples were taken 12, 24, 48, and 72 hours after establishment of each microcosm in order to monitor the subsequent abundances of the three species. We used a phase-contrast microscope at 100× to census all or a part of each subsample by means of a Palmer counting cell; all counts were converted to cells per 0.10 mL. During the census period, microcosms were provided with food semicontinuously by addition of 0.55 mg of Tetramin every 6 hours.

**Table 1:** Abundances of the three protozoan species in 0.50-mL aliquots of stock cultures (used for introduction of protozoans into the microcosms) in medium inoculated with bacteria.

	*Poterioochromonas malhamensis* (species A)	*Colpoda steinii* (species B)	Eimeriidae gen. sp. (species C)
High density	5000	1600	300
Intermediate density	100	80	50
Low density	10	10	10

### Statistical analysis

#### Community-wide analysis

We used permutational MANOVA (PERMANOVA) to test for density and sequence effects on the final community structure of the three protozoan species in microcosms on day 3 (72 hours after microcosm establishment). PERMANOVA uses an additive partitioning of a pairwise distance metric (e.g., Bray-Curtis index) according to a multifactorial ANOVA design, with significance testing by permutation to accommodate the frequent violation of the assumptions of MANOVA in community data [28]. p-values were obtained from separate sets of 999 permutations that were performed across only the pair of groups being compared, and Bonferroni correction adjusted experimentwise error rates for multiple comparison of the arrival×density treatments.

Although PERMANOVA can test for differences in the centroids of multivariate species composition between levels of a factor, we were also interested in differences in the among-plot variability in species composition (i.e., beta diversity, Anderson et al. [29]) across different levels of a factor. We performed an analysis of multivariate homogeneity of group dispersions with the nonparametric PERMDISP [30], then calculated the averaged Bray-Curtis distance of group members to the group centroid within density and sequence treatments. To examine the significance of specific treatment combinations, we used Tukey's HSD.

#### Pairwise a posteriori analysis

We tested for the effects of the identities of first-arriving and late-arriving species, density effects, and the interaction between first-arriving/late-arriving effect and density effects, using appropriate subsets of the data for pairwise a posteriori PERMANOVA. For example, the difference between the first-arrival effects of species A and B was based on a subset of sequence treatments in which either species A or species B arrived first (i.e., the first, second, fourth, and fifth columns of Fig. 1). Statistical analysis was performed in R 2.13 [31] with the community ecology package vegan [32].

## Results

**Figure 3:**
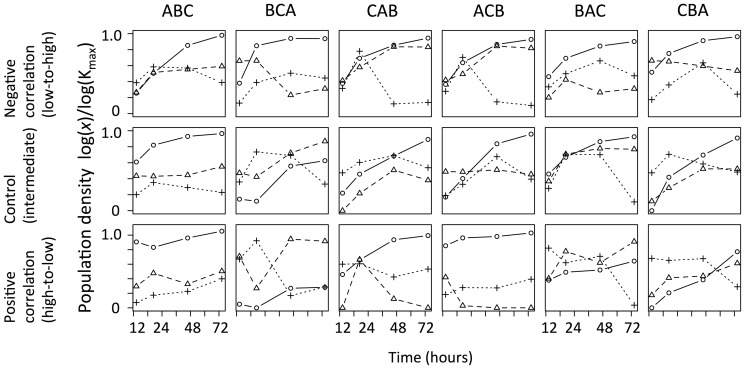
Population dynamics of the three protozoan species over 72 hours in the density (negative, control, and positive treatments; see Fig. 1) and sequence-of-introduction treatments. Densities were log_e_ (*x*+1)-transformed and then scaled to log-transformed carrying capacity for each species, and three temporal blocks were averaged. Circles, species A; triangles, species B; crosses, species C. According to Fig. 2, we used 11000/mL for species A, 2500 for species B, and 550 for species C as estimates of carrying capacity for illustrative purpose.

Direct manipulation of density and sequence in the initial 24 hours resulted in large variation in the subsequent protozoan community structure (Fig. 3). For example, in the treatment where the order of invasion was species C, then A, then last B (hereafter CAB) and the ACB treatment, crossed with positive density effects, species B was suppressed to an extremely low density or went extinct. Species A dominated numerically in 83% of the treatments but not in control×BCA or positive-density treatments crossed with BCA and BAC. Replicate communities displayed strong consistency among temporal blocks (PERMANOVA, F = 0.330, df = 2, p = 0.892), so we did not consider the effect of temporal blocks on community assembly in further analyses.

### Community-wide effects of density and sequence

**Figure 4:**
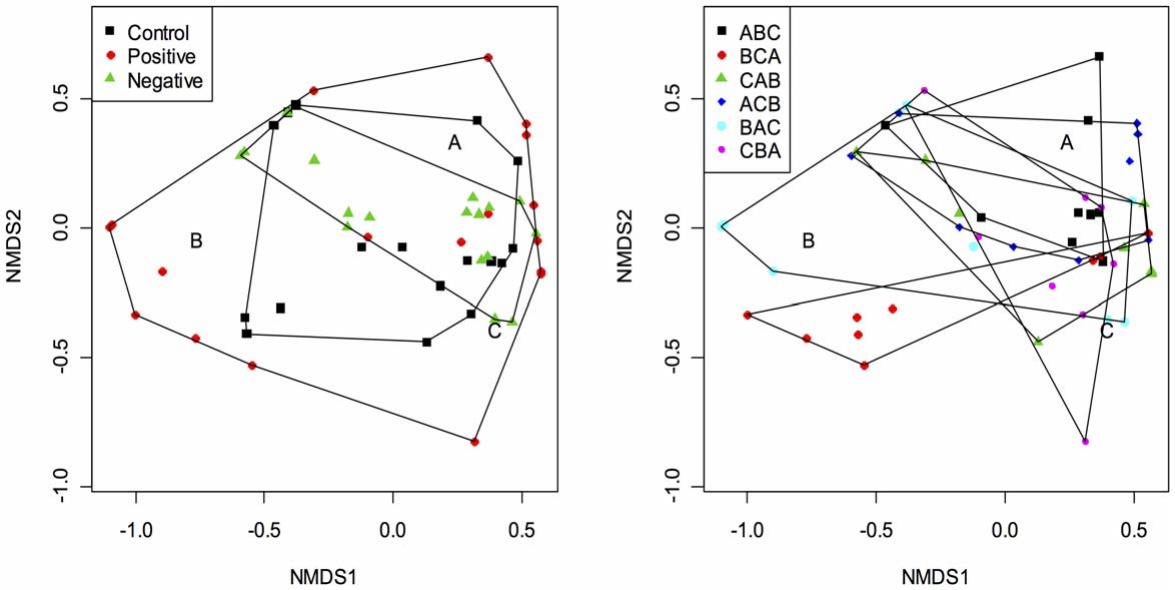
Nonmetric multidimensional scaling (NMDS) ordination plots of community composition. Left, effects of introduction density; right, effects of introduction sequence. Each symbol represents the composition of a protozoan community in an experimental microcosm, and the distance between any two points represents the difference between those two communities according to the Bray-Curtis dissimilarity metric. Letters within plots represent the locations of species A, B, and C scores in two-dimensional space. Lines represent the minimum convex hulls around the data. The stress value for the ordination was 3.29. All the combinations of the density treatments differed highly significantly in community dispersion (Table 2a). Differences in the means of community dispersion between sequence treatments were significant by Tukey's HSD significance test in the combinations of ABC/BCA, ABC/BAC, BCA/CAB, BCA/ACB, CAB/BAC, and ACB/BAC (Table 2b).

Density treatments differed significantly in average community states (PERMANOVA, F = 11.86, df = 2, p = 0.001), as did sequence treatments (F = 11.28, df = 5, p = 0.001), and notably, these two effects interacted to determine the protozoan community structure (PERMANOVA, F = 5.986, df = 10, p = 0.001). Strong density effects also caused greater dispersion in the protozoan community structure (PERMDISP, Tukey's HSD, p<0.001; Table 2, Fig. 4), but pairwise comparisons by Tukey's HSD showed that only 6 of 15 possible combinations of sequence treatments differed significantly in the levels of group community dispersion (PERMDISP, Tukey's HSD, p<0.001; Table 3, Fig. 4). Note that differences in community dispersion could be explained by interactions between density and sequence effects.

**Table 2:** Mean community dispersion (Bray-Curtis distances and corresponding p-values) from Tukey's HSD tests for comparisons of the density treatments (positive density effects, in which protozoan density in the introduced aliquots was negatively correlated with order of introduction, first high, then intermediate, then low; control, no correlation; and negative density effects, in which the density was positively correlated with the order of introduction, first low, then intermediate, then high) and (b) the sequence treatments corresponding to the six possible orders of introduction of experimental species A, B, and C into the microcosms.

	Positive	Negative
Control	0.153 (0.002)	−0.197 (<0.001)
Positive		−0.350 (<0.001)

**Table 3:** Mean community dispersion (Bray-Curtis distances and corresponding p-values) from Tukey's HSD tests for comparisons of the sequence treatments corresponding to the six possible orders of introduction of experimental species A, B, and C into the microcosms.

	BCA	CAB	ACB	BAC	CBA
ABC	0.258 (0.002)	−0.001 (1.000)	0.003 (1.000)	0.197 (0.034)	0.110 (0.516)
BCA		−0.260 (0.003)	−0.255 (0.003)	−0.061 (0.927)	−0.148 (0.196)
CAB			0.004 (1.000)	0.199 (0.041)	0.111 (0.535)
ACB				0.194 (0.039)	0.107 (0.545)
BAC					−0.088 (0.736)

#### Species pairwise comparisons

When species B was used as the first colonist (i.e., in pairwise comparisons, species B compared to A and species B compared to C), density and sequence effects interacted to determine the final community structure (Table 4). Only the main effects of density and sequence were significant when either species A or species C was the first colonist. The identity of late-arriving species caused interactive effects of density and sequence effects on the community for the combination of species A and B. In cases where either species B or species C was the late-arriving species, no main effects of density or sequence were detected, but the density-by-sequence interaction was significant (PERMANOVA, F = 3.365, df = 2, p = 0.007; Table 3). When either species C or species A was the late-arriving species, density effects primarily controlled the community structure (PERMANOVA, F = 5.578, df = 2, p = 0.003; Table 5).

**Table 4:** Pairwise a posteriori tests for (a) first arrival×density and (b) late arrival×density by PERMANOVA with Bray-Curtis distances in the protozoan community states.

	df	SS	MS	F	R^2^	p
Sequence A/B	1	1.978	1.978	33.03	0.292	0.001
Density	2	1.464	0.732	12.22	0.216	0.001
Sequence(A/B)×Density	2	1.526	0.763	12.74	0.226	0.001
Residuals	30	1.797	0.060		0.266	
Total	35	6.766			1.000	
Sequence B/C	1	1.172	1.172	11.90	0.174	0.001
Density	2	1.508	0.754	7.656	0.224	0.001
Sequence B/C×Density	2	1.206	0.603	6.121	0.179	0.001
Residuals	29	2.856	0.098		0.424	
Total	34	6.741			1.000	
Sequence C/A	1	0.315	0.315	4.808	0.107	0.008
Density	2	0.443	0.222	3.380	0.150	0.009
Sequence C/A×Density	2	0.291	0.145	2.216	0.098	0.055
Residuals	29	1.903	0.066		0.644	
Total	34	2.951			1.000	

A = *Poterioochromonas malhamensis*, B = *Colpoda steinii*, C = Eimeriidae gen. sp.

**Table 5:** Pairwise a posteriori tests for (a) first arrival×density and (b) late arrival×density by PERMANOVA with Bray-Curtis distances in the protozoan community states.

	df	SS	MS	F	R^2^	p
Sequence A/B	1	0.904	0.904	7.829	0.150	0.002
Density	2	0.633	0.316	2.741	0.105	0.034
Sequence A/B×Density	2	1.123	0.562	4.865	0.187	0.004
Residuals	29	3.348	0.115		0.557	
Total	34	6.008			1.000	
Sequence B/C	1	0.138	0.138	1.607	0.036	0.198
Density	2	0.668	0.334	3.898	0.172	0.002
Sequence B/C×Density	2	0.577	0.288	3.365	0.149	0.007
Residuals	29	2.486	0.086		0.643	
Total	34	3.870			1.000	
Sequence C/A	1	0.366	0.366	2.295	0.050	0.092
Density	2	1.779	0.890	5.578	0.243	0.003
Sequence C/A×Density	2	0.373	0.186	1.169	0.051	0.291
Residuals	30	4.784	0.159		0.655	
Total	35	7.302			1.000	

A = *Poterioochromonas malhamensis*, B = *Colpoda steinii*, C = Eimeriidae gen. sp.

In summary, whichever species arrived first had an advantage and more strongly influenced later-colonizing competitors, regardless of its initial density. A superior competitor, species B, mediated alternative community states; when arriving first, it outcompeted or suppressed the other two species, whereas it was vulnerable to extinction under the simultaneous disadvantages of late arrival and low density. In contrast, species A and C were able to overcome any disadvantage due to low initial density.

## Discussion

Our experiment and analyses demonstrated that density and sequence effects were distinct ecological mechanisms that differed qualitatively in their impacts on assembly of a three-species protozoan community and, most importantly, that density and sequence effects on assembly interacted. Density effects caused greater dispersion in the protozoan community structure without substantially changing the average community states, whereas sequence effects often altered the community states, possibly through changing the locations of the community attractors themselves (Fig. 4). Historical contingency in the protozoan community therefore arises from three sources: (1) whether or not initial densities differ sufficiently to cause density effects when the immigration sequence and times of arrival are fixed, (2) whether sequence effects determine community structure even when initial densities do not differ substantially, and (3) effects of assembly history that arise from interactions between density and sequence effects.

Although most of our experimental protozoan communities appeared to have stabilized after 72 hours (approximately 9 generations), we cannot be certain that they do not actually represent transient community states. We suggest that the durations of our experiments were generally sufficient, on the basis of the criteria presented by Grover and Lawton [33]—the intervals between invasions was longer than the generation times, the invasion interval was shorter than the time necessary for maximum population densities to develop, and the total duration of the experiment was much longer than the generation times. However, even if our final communities did not represent stable or near-stable states, theoretical work suggests that historical contingency can be important for understanding transient dynamics as well [9].

In prior studies, priority effects have often been invoked as a post hoc explanation for the observed community changes, as the investigator looked back to initial conditions to interpret current conditions [see, e.g., Robinson and Dickerson [12], Kennedy et al. [14]]. However, because early-arriving species in these studies are likely to be more abundant by the time later species arrive, the effects of sequence and density are confounded. Our work not only suggests a refined interpretation of priority effects in principle but also provides a wider framework that might be useful in decision making in practical restoration projects. For restoration ecologists, the vague concept of priority effects does not reveal when, or how many individuals of, a particular species should be introduced, because most previous studies of priority effects inherently confounded density and timing. We propose that a theory of assembly history could better guide restoration efforts if density and timing are considered separately and interactively.

Sequence effects may be characteristic of particular types of systems—they may lead us forcefully to dissect purely historical processes into trait-based mechanisms (see, e.g., Beckerman et al. [19]) for the practical purpose of gaining specific predictions about the target systems. Separating density and sequence effects can thus contribute theoretical guidance to harnessing contingency behind community assembly or at least clarifying the information demands in previous studies that have relied heavily on purely empirical, case-by-case approaches. Sequence effects may less important in other systems, however, especially over the long term: for example, Collinge and Ray [23] used a restoration project in vernal plant communities to test for historically contingent assembly but found that the order and intensity of seeding influenced plant communities only transiently, within a decade of early community formation.

An important future challenge will be to determine whether such historical forces scale up to more complex situations. Natural experiments often involve many uncontrolled variables and may require using multiple sources of information to rule out alternative hypotheses of assembly-history dynamics. Reconstructing population-genetic structure by analyzing current populations, for example, may allow us to use proxies for density effects and sequence effects of the unwitnessed past. Accumulating quantitative facts about the components of immigration history (timing, number, frequency, etc.) in island restoration, biocontrol management, and biological invasion continues to be important for understanding a large-scale imprint of assembly-history dynamics. Although our study was of a competitive community, further mechanistic lines of inquiry into assembly-history dynamics for predator-prey interactions, mutualisms, and multitrophic food webs will enrich our understanding not only of the ingredients (the species) but also of the recipes (timing and numbers of individuals) for ecological communities in an invasion-driven world.

## References

[1] Diamond JM (1975) Assembly of species communities. In: Cody ML, and Diamond JM, editors. Ecology and evolution of communities. Cambridge, MA: Harvard University Press. pp.342–444.

[2] Drake JA (1991) Community assembly mechanics and the structure of an experimental species ensemble. Am Nat 137: 1–26.

[3] Urban MC (2004) Disturbance heterogeneity determines freshwater metacommunity structure. Ecology 85: 2971–2978.

[4] Weslien J, Djupström LB, Schroeder M (2011) Long-term priority effects among insects and fungi colonizing decaying wood. J Anim Ecol 80: 1155–1162. 21569031 10.1111/j.1365-2656.2011.01860.x PMC3253338

[5] Morris WF, Wood DM (1989) The role of lupine succession on Mount St. Helens: facilitation or inhibition? Ecology 70: 697–703.

[6] Petraitis PS, Dudgeon SR (2005) Divergent succession and implications for alternative states on rocky intertidal shores. J Exp Mar Biol Ecol 326: 14–26.

[7] Houseman GR, Mittelbach GG, Reynolds HL, Gross KL (2008) Perturbations alter community convergence, divergence, and formation of multiple community states. Ecology 89: 2172–2180. 18724727 10.1890/07-1228.1

[8] Shurin JB, Amarasekare P, Chase JM, Holt RD, Hooper MF, et al (2004) Alternative stable states and regional community structure. J Theor Biol 227: 359–368. 15019503 10.1016/j.jtbi.2003.11.013

[9] Fukami T, Nakajima M (2011) Community assembly: alternative stable states or alternative transient states? Ecol Lett 14: 973–984. 21790934 10.1111/j.1461-0248.2011.01663.x PMC3187870

[10] Palmer TM, Young TP, Stanton ML (2002) Burning bridges: priority effects and the persistence of a competitively subordinate acacia-ant in Laikipia, Kenya. Oecologia 133: 372–379. 28466213 10.1007/s00442-002-1026-1

[11] Wilbur HM, Alford RA (1985) Priority effects in experimental pond communities: responses of *Hyla* to *Bufo* and *Rana* . Ecology 66: 1106–1114.

[12] Robinson JF, Dickerson JE (1987) Does invasion sequence affect community structure? Ecology 68: 587–595.

[13] Fukami T (2004) Assembly history interacts with ecosystem size to influence species diversity. Ecology 85: 3234–3242.

[14] Kennedy PG, Peay KG, Bruns TD (2009) Root tip competition among ectomycorrhizal fungi: are priority effects a rule or an exception? Ecology 90: 2098–2107. 19739372 10.1890/08-1291.1

[15] Shorrocks B, Bingley M (1994) Priority effects and species coexistence: experiments with fungal-breeding *Drosophila.* . J Anim Ecol 63: 799–806.

[16] Fukami T, Dickie IA, Wilkie JP, Paulus BC, Park D, et al (2010) Assembly history dictates ecosystem functioning: evidence from wood decomposer communities. Ecol Lett 13: 675–684. 20412280 10.1111/j.1461-0248.2010.01465.x

[17] Chase JM (2003) Community assembly: when should history matter? Oecologia 136: 489–498. 12836009 10.1007/s00442-003-1311-7

[18] Lotka AJ (1925) Elements of physical biology. Baltimore: Williams & Wilkins. 460.

[19] Beckerman A, Benton TG, Ranta E, Kaitala V, Lundberg P (2002) Population dynamic consequences of delayed life-history effects. Trends Ecol Evol 17: 263–269.

[20] Hastings A, Byers JE, Crooks JA, Cuddington K, Jones CG, et al (2007) Ecosystem engineering in space and time. Ecol Lett 10: 153–164. 17257103 10.1111/j.1461-0248.2006.00997.x

[21] Young TP, Chase JM, Huddleston RT (2001) Community succession and assembly: comparing, contrasting and combining paradigms in the context of ecological restoration. Ecol Rest 19: 5–18.

[22] Connell JH, Slatyer RO (1977) Mechanisms of succession in natural communities and their role in community stability and organization. Am Nat 111: 1119–1144.

[23] Collinge SK, Ray C (2009) Transient patterns in the assembly of vernal pool plant communities. Ecology 90: 3313–3323. 20120801 10.1890/08-2155.1

[24] Miller TE, Kneitel JM (2005) Inquiline communities in pitcher plants as a prototypical metacommunity. In: Holyoak M, Leibold MA, Holt RD, editors. Metacommunities: spatial dynamics and ecological communities. Chicago: University of Chicago Press. pp. 122–145.

[25] Miller TE, terHorst CP (2012) Testing successional hypotheses of stability, heterogeneity, and diversity in pitcher-plant inquiline communities. Oecologia in press. 10.1007/s00442-012-2292-1 22430372

[26] Miller TE, Kneitel JM, Burns JH (2002) Effect of community structure on invasion success and rate. Ecology 83: 898–905.

[27] Schaffer WM (1981) Ecological abstraction: the consequences of reduced dimensionality in ecological models. Ecol Monogr 51: 383–401.

[28] Anderson MJ (2001) A new method for non-parametric multivariate analysis of variance. Aust Ecol 26: 32–46.

[29] Anderson MJ, Crist TO, Chase JM, Vellend M, Inouye BD, et al (2011) Navigating the multiple meanings of β diversity: a roadmap and compass for the practicing ecologist. Ecol Lett 14: 19–28. 21070562 10.1111/j.1461-0248.2010.01552.x

[30] Anderson MJ, Ellingsen KE, McArdle BH (2006) Multivariate dispersion as a measure of beta diversity. Ecol Lett 9: 683–693. 16706913 10.1111/j.1461-0248.2006.00926.x

[31] R Development Core Team (2011) R: a language and environment for statistical computing. Vienna, Austria: R Foundation for Statistical Computing.

[32] Oksanen J, Blanchet FG, Kindt R, Legendre P, O'Hara RB, et al. (2011) vegan: Community Ecology Package. R package version 1.17-12. http://CRAN.R-project.org/package=vegan.

[33] Grover JP, Lawton JH (1994) Experimental studies on community convergence and alternative stable states: comments on a paper by Drake et al. J Anim Ecol 63: 484–487.

